# Sleep disorders among women with post-menopausal osteoporosis

**DOI:** 10.1192/j.eurpsy.2024.1615

**Published:** 2024-08-27

**Authors:** A. Feki, I. Sellami, B. Trabelsi, Z. Gassara, S. Ben Djemaa, A. Abbes, M. Ezzeddine, M. H. Kallel, H. Fourati, R. Akrout, Y. Mejdoub, S. Baklouti

**Affiliations:** ^1^Rheumatology; ^2^Occupational medicine, Hedi Chaker Hospital; ^3^ Medicine university; ^4^Preventive medicine, Hedi Chaker Hospital, Sfax, Tunisia

## Abstract

**Introduction:**

Osteoporosis (OP) is characterized by low bone mass and microarchitectural deterioration of bone tissue. Recent studies have suggested that sleep may significantly influence the pathophysiology of OP.

**Objectives:**

In the present study, we aimed to determine sleep disorders among women with post-menopausal OP.

**Methods:**

A cross-sectional study was conducted between January and June 2023. Patients with post-menopausal OP who visited the rheumatology department in a university hospital in Tunisia were interviewed. The Pittsburgh Sleep Quality Index (PSQI). It is a seven-component scale, including: sleep quality (C1), sleep latency (C2), sleep duration (C3), sleep efficiency (C4), sleep disturbances (C5), sleep medication use (C6), and daytime dysfunction (C7).PSQI ≤ 7 indicated normal sleep quality, and PSQI > 7 indicated poor sleep quality.

**Results:**

Ninety-three women diagnosed with post-menopausal OP were interviewed. the number of complete questionnaires was 72. The valid rate was 77.4%. All were women. The mean age was 72.5 (±1.08). The median duration of menopause was 23 years (IIQ= [10.5-28.5]). Forty-five women were diagnosed with bone fractures (62%). Thirty-three patients (45.8%) were obese (IMC>30). The median PSQI score was 16 (IIQ = [6-18]). Forty-seven participants (65.3%) had poor sleep quality (PSQI > 7). According to the items of PSQI: the median score of sleep duration, sleep Efficiency and sleep disturbances was 1 (IIQ= [1 -2]) for each item. The median score of sleep latency was 3 (IIQ=[2-3]). For daytime dysfunction, the median score was 2 (IIQ=[0-3]).

Study analytics revealed a significant association between daytime dysfunction and the presence of bone fractures (p=10^-3^), the same was with sleep disturbances and bone fractures (p=10^-3^). Body mass index (BMI) was significantly and inversely associated with sleep quality (r= -0.313; p= 0.007). Sleep latency was significantly associated with physical activity (p<10^-3^).

**Conclusions:**

In conclusion, our results suggest that sleep quality is associated with physical activity and BMI. This is consistent with the most recent evidence in the literature. These findings support expanding the scope of wellness programs to promote healthy sleep among osteoporotic women.

**Disclosure of Interest:**

None Declared

